# In vivo antimalarial evaluation of some 2,3-disubstituted-4(3*H*)-quinazolinone derivatives

**DOI:** 10.1186/s13104-015-1578-x

**Published:** 2015-10-20

**Authors:** Yihenew Simegniew Birhan, Adnan Ahmed Bekhit, Ariaya Hymete

**Affiliations:** Department of Chemistry, Natural and Computational Sciences College, Debre Markos University, P.O. Box 269, Debre Markos, Ethiopia; Department of Pharmaceutical Chemistry, Alexandria University, Alexandria, 21215 Egypt; Department of Pharmaceutical Chemistry and Pharmacognosy, School of Pharmacy, Addis Ababa University, P.O. Box 1176, Addis Ababa, Ethiopia

**Keywords:** Quinazolinones, Malaria

## Abstract

**Background:**

Malaria is a neglected tropical parasitic disease affecting billons of people around the globe. Though the number of cases and deaths associated with malaria are decreasing in recent years, it is the most deadly disease in the world. This study aimed at investigating the in vivo antimalarial activities of some 2,3-disubstituted-4(3*H*)-quinazolinone derivatives.

**Results:**

The in vivo antimalarial activities of the test compounds (**6**–**9** and **11**–**13**) were investigated using the 4-day suppressive standard test in mice infected with chloroquine-sensitive *Plasmodium berghei* ANKA strain. The tested compounds showed significant antimalarial activities with mean percentage suppression of 43.71–72.86 % which is significantly higher than the negative control group (p < 0.05). Compounds **12** and **13** displayed better antimalarial activities from the group with mean percentage suppression of 67.60 and 72.86 % respectively.

**Conclusion:**

The tested compounds showed significant in vivo antimalarial activities in mice infected with *P. berghi* ANKA strain. Thus, 3-aryl-2-(substitutedstyryl)-4(3*H*)-quinazolinones represent a possible scaffold for the development of antimalarial agents.

## Background

Malaria is a neglected tropical parasitic disease caused by a *Plasmodian* protozoan species [1]. It is transmitted by the bite of female *Anopheles* mosquito through injection of sporozoites at the time of blood suck [2]. In 2012, there were an estimated 3.4 billion people at risk of malaria, of whom 1.2 billion are at high risk. Children, pregnant women, HIV patients and travelers to sub-Saharan African countries are at increased risk of severe malaria, if infected with *P. falciparum* [3]. The global number of malaria cases and deaths were estimated to have decreased since 2005 due to the expansion of access to rapid diagnostic tests, use of long-lasting insecticidal nets (136 million in 2013), quality assured artemisinin combination therapy (ACT) (331 million courses were produced in 2012) and increased funding for malaria control programs (US$ 2.5 billion in 2012) [4, 5].

Artemisinins and ACT with fixed dose combinations are the first-line treatment for *falciparum* malaria in almost all regions where malaria is endemic [6, 7] due to the emergence of resistance *P. falciparum* strains to the older drugs [8–10]. Although artemisinins are potent and rapidly acting antimalarial drugs, their widespread use for treating patients with *P.falciparum* malaria raises the question of emerging drug resistance [11]. Research findings revealed that treatment failures to artesunate-amodiaquine (AS-AQ) and artemether-lumefantrine (ALU) has been observed in different parts of the globe [12, 13]. In addition, artemisinin resistance has already emerged along the Thai-Cambodian border [14, 15]. Taking this into account, an aggressive and ambitious global effort is being made to discover new effective drugs for the treatment of malaria [16, 17].

The quinazoline nucleus, a basic unit found in various naturally occurring bioactive alkaloids, have continued to attract interest due to their diverse pharmacological activities [18–21]. Different reports revealed that a wide number of quinazolinone derivatives demonstrated promising antimalarial activities [20, 22]. Recently, our group has synthesized and tested the antileishmanial activities of some 2,3-disubstituted-4(3*H*)-quinazolinone derivatives [23]. The aim of the present study was to evaluate the in vivo antimalarial activities of these compounds (**6**–**9** and **11**–**13**) using Swiss albino mice infected with chloroquine-sensitive *P. berghei* ANKA strain. In addition, oral acute toxicity tests were performed for compounds with significant antimalarial activities.

## Methods

### Chemicals and reagents

Absolute ethanol, absolute methanol, distilled water, iodine, Giemsa stain, Tween 80, 1 % gum acacia were used in the study.

### Experimental animals and test strains

Swiss albino male mice (age 6–8 weeks and weight 20–32 g) bred and maintained under standard conditions (temperature of 22 ± 3 °C, relative humidity of 40–50 % and 12 h light/12 h dark cycle), with food and water ad libitum in the animal house of Biomedical Laboratory, Department of Biology, Faculty of Sciences, Addis Ababa University (AAU). They were acclimatized for one week for the experimental conditions.

Chloroquine-sensitive *P. berghei* ANKA strain used to infect the mice for a 4-day suppressive test was obtained from Biomedical Laboratory, Department of Biology, Faculty of Sciences, AAU. The parasite was maintained by serial passage of blood from infected mice to non-infected ones on weekly basis.

### Reference drugs

For the in vivo antimalarial activity testing, chloroquine phosphate (EPHARM, Addis Ababa, Ethiopia) was used as a reference drug.

### In vivo *a*ntimalarial activity test

#### Parasite inoculation

Swiss albino mice previously infected with *P. berghei* and having parasitemia level of 20–30 % were used as donors. The donor mice was then sacrificed by decapitation and blood was collected by cardiac puncture into heparinized vacutainer tube containing 0.5 % trisodium citrate. The blood was then diluted with physiological saline based on parasitemia level of the donor mice and the red blood cell (RBC) count of normal mice, in such a way that 1 ml of blood contains 5 × 10^7^ infected RBCs. Each mouse was given 0.2 ml of diluted blood intrapreritoneally, which contained 1 × 10^7^*P. berghei* infected RBCs [24].

### Drugs used

Both chloroquine phosphate and the test compounds (**6**–**9** and **11**–**13**) were dissolved in 70 % Tween 80 and 30 % ethanol. These solution were further diluted tenfold with distilled water to result in stock solutions containing 7 % Tween 80 and 3 % ethanol.

### Grouping and dosing of animals

For the antimalarial evaluations of the target compounds, infected mice were randomly divided into nine groups of five mice per cage. Group **1** served as a negative control and group **2** served as a positive control. A vehicle containing a solution of 7 % Tween 80, 3 % ethanol and 90 % distilled water (2 ml/100 g) and chloroquine phosphate 25 mg/kg (48.46 µmol/kg) was administered orally to group **1** and **2** respectively. The remaining groups (group **3**–**9**) were treated with equimolar amounts (48.46 µmol/kg) of the synthesized compounds through oral route for four consecutive days [25].

For the oral acute toxicity studies of each compound (**12** and **13**), 36 male Swiss albino mice (approximately 20 g each) were randomly assigned to six groups (containing six mice per group). Group **1**–**5** were treated with each compound suspended in a vehicle containing 1 % gum acacia at a dose of 10, 50, 100, 200 and 300 mg/kg, respectively. The sixth group received vehicle containing 1 % gum acacia (served as a negative control group) at a maximum dose of 1 ml/100 g of body weight by oral route [23].

### The standard 4-day suppressive test

The 4-day standard suppressive test was used to evaluate the in vivo antimalarial activities of the test compounds using *P. berghei* infected mice [26]. Infected mice were randomly divided into their respective group as described under grouping and dosing. Treatment was started 2 h after mice had been inoculated with the parasite and continued for four consecutive days. Twenty-four hours after the last treatment (5th day), blood smears were taken from the tail of all mice, air dried, fixed with absolute methanol and stained with 6 % Giemsa stain. The parasitemia and percentage inhibition were then determined microscopically by counting four fields of approximately 100 erythrocytes per field. The efficacies of compounds were finally assessed by comparison of blood parasitemia and mouse survival time in treated and untreated control mice [27].

### Parasitemia measurement

Thin blood smears were made from the tail of each mouse on the 5th day. The smears were applied on microscope slides (76 × 26 mm) (Menzel-Glaser, Germany), fixed with absolute methanol and stained with 10 % Giemsa stain at pH 7.2 for 15 min. The stained slides were washed gently using distilled water and air dried at room temperature. Two stained slides for each mouse were examined under Olympus microscope (CHK2-F-GS, Taiwan) with an oil immersion nose piece of 100× magnification. Four different fields on each slide were examined to calculate the average parasitemia as shown below [28].

$$ {\text{Percentage parasitemia }} = \frac{\text{Number of parasitized RBC}}{\text{Total number of RBC }}  \times { 1}00 $$

Finally, percentage parasitemia suppression of the synthesized were compared with respect to the controls and percentage suppression was calculated using the following formula [29]

$$ {\text{Percentage suppression }} = \frac{{{\text{Mean parasitemia of negative control }} - {\text{Mean parasitemia of treated group}}}}{\text{Mean parasitemia of negative control group}} \times 100 $$

### *In vivo a*cute toxicity test

The oral acute toxicities of the test compounds (**12** and **13**) with promising antimalarial activities were investigated in a dose of 10, 50, 100, 200 and 300 mg/kg. Animals were observed for gross body changes such as loss of appetite, hair erection, lacrimation, convulsions, salivation, diarrhea, mortality and other signs of overt toxicity [23].

### Ethical clearance

The care and handling of the experimental animals was according to international guideline for use and maintenance of experimental animals [30] and Addis Ababa University, School of Pharmacy Ethics committee approved the protocol.

### Statistical analysis

Results of the study were expressed as mean ± standard deviation and statistical significance for suppressive test was determined by one-way ANOVA using Origin 6.0 software. Data on survival time, percentage parasitemia and percentage suppression was analyzed using Microsoft office excel 2007. All data was analyzed at 95 % confidence interval.

## Results and discussion

### In vivo antimalarial activity results

The in vivo model was employed for this study because it takes into account the possible pro-drug effects and possible involvements of immune system in eradication of infection [31]. *P. berghei* ANKA stain was used in the prediction of treatment outcomes [32] and hence it was an appropriate parasite for the study. The standard 4-day suppressive test, which mainly evaluates the antimalarial activity of candidate drugs on early infections, is commonly used for antimalarial screening. In this method, determination of percentage inhibition of parasitemia is the most reliable parameter. The mean parasitemia level ≤90 % relative to that of placebo-treated control animals usually indicates that the test compound is active in standard screening studies [33]. In this study, the standard 4-day suppressive test was used to evaluate the antimalarial activities of the synthesized compounds on chloroquine-sensitive *P. berghei* infected mice. Equimolar amounts (48.46 µmol/kg) of the synthesized compounds and the standard drugs were administered through the oral route. The percent suppression, percent parasitaemia, and mean survival time of the mice treated with the synthesized compounds were compared against the control groups, as shown in Table 1.

**Table 1:**
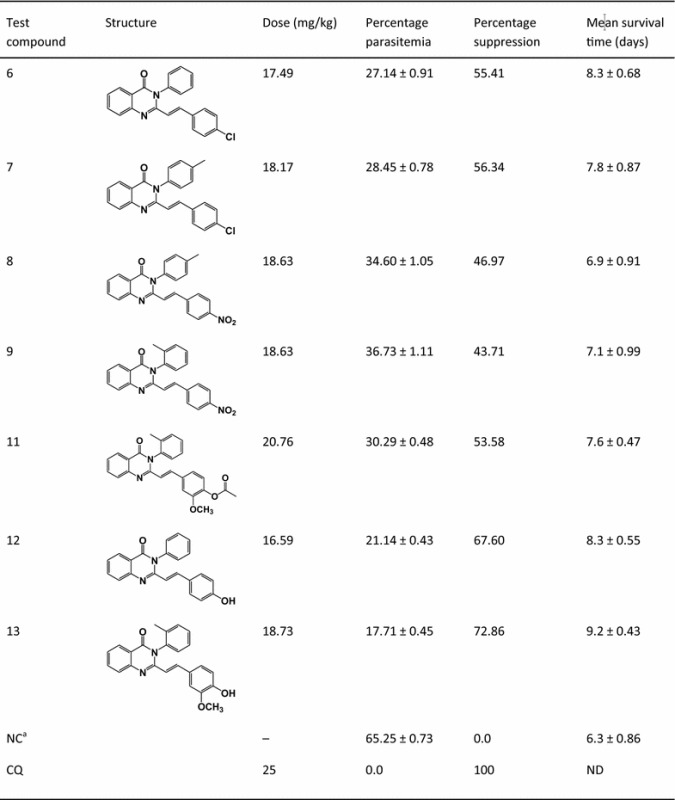
Data for antimalarial activity testing of the synthesized compounds (48.46 µmol/kg)

*NC* negative control, *CQ* chloroquine phosphate, *ND* no death

^a^Values are Mean ± SD, P < 0.05

The percentage parasitemia determined for all test compounds were significantly low relative to the negative control (p < 0.05), showing that the compounds are active. This significant antimalarial activity of the target compounds was also in agreement with that of the activity of 4(3*H*)-quinazolinone derivatives on the same parasite strain [34]. The test compounds, **6**, **7, 11, 12**, and **13** displayed mean percentage suppression of greater than 50 %. On the other hand, compounds **8** and **9** had less than 50 % mean percent suppression compared to the untreated group (Table 1).

Compound **13** was the most active of the tested compounds with mean percentage suppression of 72.86 %. The mean parasitemia level in mice treated with **13** (17.71 ± 0.45) was found to be approximately four times lower than the negative control (65.25 ± 0.73), showing the compound has greatly reduced the parasite load. This significant activity was further supported by better mean survival time (9.2 ± 0.43) of mice compared with other test compounds but less than those of positive control (chloroquine-treated) group that did not show any death during the experimental period. Compound **12** displayed the next significant (p < 0.05) antimalarial activity with percentage suppression of 67.60 % that is further confirmed by mean survival time (8.3 ± 0.55) (Table 1). The highest suppression effect of **12** and **13** may be attributed to the presence of polar groups [hydroxyl group (–OH) in both **12** and **13**, methoxy group (–OCH_3_) in **13**] at 2-styryl moiety that may interact through hydrogen bonding with the active site.

The remaining compounds; **6**, **7** and **11** demonstrated moderate antimalarial activities with mean percentage suppression of 55.41, 56.34 and 53.58 % respectively. These compounds possess electron withdrawing group at *para* position of 2-styryl group, which may increase the likelihood of strong hydrophobic interaction between the compounds and the active sites. However, they displayed relatively lower antimalarial activities as compared to compound **12** and **13**.

### Acute toxicity results

The acute toxicity study indicated that compounds **12** and **13** caused no mortality in all doses (50, 100, 200 and 300 mg/kg) within the first 24 h as well as for the following 14 days. Physical and behavioral observations of the experimental mice also revealed no visible signs of overt toxicity. Thus, compound **12** and **13** showed no inherent acute toxicity signs at a maximum dose of 300 mg/kg.

## Conclusions

The antimalarial activities of some 3-aryl-2-(substitutedstyryl)-4(*3H*)-quinazolinone derivatives were tested. All the tested compounds showed significant antimalarial activities as compared to the negative control group (p < 0.05). Better antimalarial activities were observed for compound **12** and **13** with mean percentage suppression of 72.86 and 67.60 % respectively. Thus, 2,3-disubstituted-4(3*H*)-quinazolinines containing an aromatic substitution at 3-position and substitutedstyryl moiety at 2-position represent a possible scaffold for the development of new antimalarial agents.

## Abbreviations

ACT: artemisinins combination therapy
AS: artesunate
AQ: amodiaquine
A: artemether
LU: lumefantrine
RBC: red blood cells
AAU: Addis Ababa University
EPHARM: Ethiopian Pharmaceuticals manufacturer
